# Auction mechanism on construction land quota with selection on land location

**DOI:** 10.1371/journal.pone.0263075

**Published:** 2022-01-25

**Authors:** Yang Deng, Weidong Meng, Bo Huang, Jingyu Liu

**Affiliations:** School of Economics and Business Administration, Chongqing University, Chongqing, China; LUMSA: Libera Universita Maria Santissima Assunta, ITALY

## Abstract

Due to unreasonable pricing, farmers have low enthusiasm for reclaiming their homesteads, which can be used to generate construction land quota. This paper studies how to design a feasible pricing mechanism to stimulate the enthusiasm of farmers. First, we analyze the practice that the local government gives the developers with quota the selection, the right to select the location of the land to be auctioned. Then, applying sequential auction theory, we propose first- and second-price sealed-bid sequential auction models and design quota auction pricing mechanisms. Through theoretical and numerical analysis, we obtain the equilibrium strategy and analyze the impact of selection and on developers’ bidding pricing on quota. The results show that the selection can enhance the developer’s quota bidding price and farmers’ income. And the higher the value of selection to the developer, the higher the quota bidding price and farmer’s income. Contrarily, the larger the number of developers, the smaller the quota bidding price and farmers’ income. Finally, the quota bidding price and farmers’ income in the second-price sealed-bid sequential auction are higher than in the first-price sealed-bid sequential auction.

## Introduction

In order to ensure sufficient arable land for cultivating food, the Chinese central government has implemented a construction land quota system to limit the amount of cultivated land developed into construction land by local governments. Under this system, the central government grants a certain number of construction land quotas to local governments every year as planned, and each local government can only develop cultivated land within this specified quota. With the rapid advancement of China’s urbanization process, the demand for cultivated land development in cities has increased rapidly [1]. Although there are sufficient cultivated land resources in various regions, the central government plans to allocate scarce quotas. This can result in local governments having no right to develop arable land into construction land. A large number of development projects can thus be delayed or even canceled, hindering the progress of urbanization. At the same time, urbanization has attracted many people from rural areas into the cities, resulting in rural homesteads being left idle and causing a wastage of rural land resources.

To solve this problem, Chongqing, Chengdu, and other cities, as well as a large number of domestic follow-up cities, have innovated the construction land quota system. Under this improved system, the local government encourages farmers to voluntarily withdraw from the homestead, and professional companies dismantle the buildings on the idle homestead and reclaim it (i.e., rural construction land) into cultivated land. Since the local government has turned rural construction land into cultivated land, the same area of cultivated land in the city can be developed into construction land [2–4]. In this manner, governments have obtained supplementary construction land quotas in addition to those allocated by the central government. In order to encourage farmers to reclaim, the local government sells the construction land quotas generated by the reclamation to developers in need, and the subsequent income belongs to the farmers.

Although the additional quotas of reclaimed construction land can alleviate the problem of insufficient quotas of construction land, the local government adopts the cost plus pricing method based on reclamation cost. This is unreasonable as the resulting price is too low, which has affected farmers’ enthusiasm for reclamation. [5–8]. Such unreasonable pricing has been pointed out by domestic scholars [9–13]. Xu [14] combined the two stages of quota production and use, and tried to use the cost and surplus methods to build a quota price calculation model from the perspective of cost and income. The authors used the relevant data of Dezhou City and Jimo City for empirical calculations. Meanwhile, Chen et al. [15] improved the pricing method of Qiu and Qiu [16], taking the view that the transaction price of construction land quota should include the cost and market prices. Thus, they put forward the market-oriented transaction price system. Wen and Zhang [17] argued that the price of construction land quota should be the price of land for construction purposes. Feng et al. [18] and Liu et al. [19] posit that because the construction land quota trading market is mainly controlled by the local government rather than the market, its price hardly reveals the true value of the quota. These studies point out that the quota price should include the use price, the market price, and the price of land for construction. However, these studies have not yet fully understood the true value of this construction land quota, and have failed to provide effective suggestions on the pricing mechanism. The real value of the quota is manifested as that the undeveloped urban farmland can be developed as construction land immediately. Therefore, the value of the quota is an opportunity cost value, which is the developers’ private information. This study holds that the sealed auction mechanism is one of the best ways to reveal the true value of the construction land quota. According to the practice transaction of construction land quota, only by having both quota and construction land can the land be developed. Therefore, it is more reasonable to use the two-stage sequential sealed auction mechanism.

Sequential auction theory is applied in reality to reveal the rationality of the real value of goods [20–24]. Milgrom [25] solved the transaction pricing problem of radio spectrum in the United States by determining the true value of radio spectrum through the participation of bidders in the auction. De Silva et al. [26] applied the auction to the construction contract, and also considered the synergy between the projects. Finally, they argued that winners of early auctions are more likely to participate in later auctions. The authors provide evidence to support this prediction through continuous building auctions conducted by the Oklahoma Department of Transportation. Branco [27] and adopted two-stage sequential auctions, and believed that there was no fierce competition in the first stage, which created an advantage for the second stage auction and thus reduced the expected price. The latter, however, Menezes and Monteiro [28] came to the opposite conclusion that expected prices rose instead of falling. Leufkens and Peeters [29] compared the two sequential auction forms in the case of two bidders and believed that the first-price sealed-bid sequential auction was superior to the second-price sealed-bid auction. Leufkens et al. [30] also compared two sequential auctions with three different scales of synergies in the case of four bidders through a series of experimental methods. The authors believed that the first-price sealed-bid sequential auction with small and medium-sized synergies in regular procurement auctions was superior to the second-price sealed-bid sequential auction; however, when the synergies were very large, the two sequential auctions were very similar. These above studies have carried out in-depth examinations on sequential auction and compared the differences between the first- and second-price sealed-bid sequential auction. However, there are few studies on the application of sequential auction method to the quota pricing mechanism.

Considering the practice that the local government gives the developers with quota the selection, the right to choose land to be auctioned. It is believed that the value of the construction land selected by developers is reflected by whether they have the right to select [31]. Wang and Yang [32] think that the construction land quota contains selection prices and that it is associated with the degree of selection prices and the economy’s development: developers in more developed regions are willing to pay higher prices for selection. Meanwhile, Wu [33] believes that the selection saves the time value of the development of construction land, and realizes the combination of the owner of the quota and the user of the construction land. This is also the main reason why Liu et al. [34] believes that there is a time lag and low efficiency in the dispersion of urbanization in China. Lu [35] defined the income obtained from the quota selection of construction land as the selection income. The author constructed a mathematical model of the developers’ participation quota and land auction and studied the existing problems in the construction land quota system. These studies highlight the importance of construction land quota selection for quota pricing. However, they have not made relevant research on how to price quotas when they have the right of select, and how the right of select affects the pricing of quotas.

In order to solve the problem caused by the insufficient construction land quota allocated by the central government, local governments encourage farmers to reclaim their idle homesteads to generate additional construction land quotas and sell them to developers in need. Consequently, to boost farmers’ enthusiasm for reclamation, the government uses auctions to sell the reclaimed construction land quota. At the same time, to increase the value of this indicator to developers, as well as the developer’s quotation, the local government gives the right of selecting the location of the land to be auctioned to the developer who has bought construction land quota from the government. In other words, the developer can select the land that best meets its needs from among the multiple the multiple alternative land provided by the government the multiple alternative land, and thereafter participate in its auction.

The remainder of this study is organized as follows. Section 2 introduces the problem and variables. Section 3 obtains and analyses the equilibrium solutions. Section 4 presents the numerical analysis to further verify the model and proposition. Section 5 presents the conclusions and policy suggestions.

## Problem and variables

### Problem

In order to solve the problem caused by the insufficient construction land quota allocated by the central government, local governments encourage farmers to reclaim their idle homesteads to generate additional construction land quotas and sell them to developers in need. Consequently, to boost farmers’ enthusiasm for reclamation, the government uses auctions to sell the reclaimed construction land quota. At the same time, to increase the value of this indicator to developers, as well as the developer’s quotation, the local government gives the right of selecting the location of the land to be auctioned to the developer who has bought construction land quota from the government. In other words, the developer can select the land that best meets its needs from among the multiple the multiple alternative land provided by the government the multiple alternative land, and thereafter participate in its auction.

A local government is preparing to auction a piece of urban construction land. However, it lacks a quota for construction land. Therefore, the government encourages farmers to voluntarily reclaim the rural vacant homesteads into cultivated land to produce the construction land quota and through the sealed auction method to sell to the need of developers. Meanwhile, the government stipulates that the developer who has obtained the quota in the first-stage auction has the selection to select the intended plot as the construction land in the second-stage auction, within the scope of urban planning and general land use planning. Then the government holds a second auction to sell the selected construction land to developers who also participated in the first auction. Moreover, the project can only be developed when the developer is able to acquire the construction land quota and the construction land simultaneously.

Obviously, the value of the right of select is mainly reflected in the select of the expected construction land [36]. Because the developer who wins the construction land quota can select the prospective land. On the contrary, the developer who fails to win the quota cannot select the prospective land. Therefore, developers who fail to win the quota value the land to be auctioned at a lower price than before. The main reason for the lower than the original evaluation is that the matching degree of the land is reduced. The higher the mismatch degree, the greater the degree of land discount, and the greater the value of the selection for developers. Thus, all developers are willing to pay a higher price for the construction land quota.

### Variables

According to China’s construction land quota system, the government can sell the construction land quota and construction land by auction. The developer who wins the construction land quota by auction has the right to choose the construction land. The use of the selection improvers the match between the land chosen by the developer and the construction land expected to be developed; that is, the developer can obtain higher utility [37]. Therefore, we establish a sequential auction model for construction land quota and construction land based on the Hedonic utility theory. The basic assumptions and auction process are as follows:

- Assume that the risk-neutral seller (the Chinese government) sells two items by holding two successive auctions. The first (second) stage is the construction land quota (construction land) auction. Furthermore, the construction land quota and the construction land value are not correlated.
- Assume that the total number of bidders is *n*. Bidder *i*∈{1,2,…,*n*} has a utility function *u*:*R*_+_→*R*, which satisfies *u*(0) = 0,*u*’>0,*u*"<0.Each bidder pursues utility maximization and has independent private value. One may assume that bidder *i*’s valuation of the construction land quota is *v*_1*i*_ and that of the construction land is *v*_2*i*_. These independently obey the uniform distribution on [0,*ε*] and [0,1] respectively, where *ε*<1, the distribution function is *F*_*i*_(⋅), and the density function is *f*_*i*_(⋅).
- *n* risk-neutral bidders participate in the two stages of auction simultaneously and successively. The winner in the first stage holds the construction land quota, and can choose the land in line with the urban, rural, and land planning as the land for their own construction project. Furthermore, they can participate in the second stage of commodity auction. Therefore, the loser *F* in the first stage has a land depreciation coefficient $r_{L,F_{j}}\in [0,1]$ for the construction land. The land depreciation coefficient $r_{L,F_{j}}$ is due to the existence of the quota selection of the construction land. This makes the construction land auctioned in the second stage less matched with the construction land expected to be developed by the developer without selection. Therefore, land depreciation occurs. The smaller $r_{L,F_{j}}$ is, the lower the degree of matching between the construction land auctioned in the second stage and the construction land expected to be developed by the loser in the first stage. Furthermore, the higher the land depreciation will be for the developers who have no selection. Therefore, all developers are more willing to obtain the selection to obtain the competitive advantage in the second stage. Further, assume that the willingness to pay for the loser in the first stage is $v_{2F}=r_{L,F_{j}}v_{2F}$. The willingness to pay *v*_2*L*_ for the winner of the first stage of obtaining construction land remains unchanged. Finally, the winner with the highest bid in each stage wins the goods in this stage.

Table 1 summarizes the parameter symbols and meanings involved in this model.

**Table 1:** Summary of parameter symbols and meanings.

Symbols for quantities	meaning
*i*	The developer *i* = 1,2,…,*n* represents *n* bidders.
*L*	The winner of the first auction is the developer with the selection.
*F*	The losers in the first stage auction are the developers who have no selection.
*v*_1*i*_	The developer’s valuation of the construction land quota in the first stage auction.
*v*_2*i*_	The developer’s valuation of the construction land in the second stage auction.
$r_{L,F_{j}}$	Coefficient of land depreciation.
$\Delta (r_{L,F_{j}})$	Avoid land depreciation caused by the loss of the right to choose and the reduction of the degree of matching of construction land.
*b*_*i*_	Quotation from the developer.
*ε*	The upper limit of valuation of construction land quotas.
*β* ^*I*^	The first-price sealed-bid sequential auction under the developer equilibrium bid.
*β* ^*II*^	The second-price sealed-bid sequential auction under the developer equilibrium bid.
*E*(*R*)	Expected income of construction land quota, namely the amount of farmers’ compensation.

## Equilibrium solutions

### Equilibrium bidding price of the first-price sealed-bid sequential auction

The reverse recursion method is used to solve the developers’ balanced quotation strategy for construction land quotas and construction land in the first-price sequential auction. Then, Proposition 1 is obtained as follows:

#### Proposition 1

For the first-price sealed-bid sequential auction format, the developers’ equilibrium biding strategies in the two stages are as follows:

$$\beta_{1i}^{I}(v_{1i})=\frac{n-1}{n}v_{1i}+\Delta^{I}(r_{L,F_{j}})$$

$$\left\{ \begin{array}{l} \beta_{{}_{2L}}^{I}(v_{2L})=\frac{n-1}{n}v_{2L}\\ \beta_{{}_{2F_{j}}}^{I}(v_{2F_{j}})=\frac{n-1}{n}r_{L,F_{j}}v_{2F_{j}},j=1,2,\ldots ,n-1 \end{array}\right.$$

where, $\Delta^{I}(r_{L,F_{j}})=\frac{1}{2n}(1-r_{L,F_{j}}{}^{2})$, $0\leq r_{L,F_{j}}\leq 1$.

Proof: Consider the second stage of the first-price sequential auction. The construction land quote is unrelated to the value of the land. Therefore, the quotation of the second stage does not depend on the transaction price of the first stage, but only depends on the bidder’s value. Suppose there is an equilibrium in the second-stage auction, where the bidder pays the price *b*_2*i*_ and the bidder of type *S* = {*L*,*F*_1_,*F*_2_,…,*F*_*n*−1_} uses strategy *β*_*S*_. Further assume that *β*_*S*_ is an increasing and differentiable function with an inverse function *ϕ*_*S*_ = (*β*_*s*_)^−1^. Obviously, there is $\beta_{L}(0)=\beta_{F_{j}}(0)=0$, *j* = 1,2,…,*n*−1. Then, we can say $\bar{b}=\beta_{L}(1)=\beta_{F_{j}}(1)$, which represents the highest bid jointly submitted by the bidders. If the risk is neutral and the utility function is linear, the winner *L* in the first stage obtains the net utility *u*(*b*_*L*_) = *v*_*L*_−*b*_*L*_ from the construction land. Then, the utility function of bidder *L* is:

$$U_{2L}(b_{2L})=(v_{2L}-b_{2L})b_{2L}^{n-1}$$ (1)

Take the partial derivative of the utility function to obtain the first-order condition:

$$\frac{\partial U_{2L}}{\partial b_{2L}}=-b_{2L}{}^{n-1}+(v_{2L}-b_{2L})(n-1)b_{2L}{}^{n-2}$$ (2)

Utility maximization makes the first partial derivative zero to obtain the following:

$$b_{2L}=\frac{n-1}{n}v_{2L}$$ (3)

Similarly, we find that the net utility of the construction land obtained by the loser *F*_*j*_ in the first stage bidding is $u(b_{2F_{j}})=r_{L,F_{j}}v_{2F_{j}}-b_{2F_{j}}$, and the utility function of the bidder *F*_*j*_ is:

$$U_{2F_{j}}(b_{2F_{j}})=(r_{L,F_{j}}v_{2F_{j}}-b_{2F_{j}})b_{2F_{j}}{}^{n-1}$$ (4)

Take the partial derivative of the utility function to obtain the first-order condition:

$$\frac{\partial U_{2F_{j}}}{\partial b_{2F_{j}}}=-b_{F_{j}}{}^{n-1}+(r_{L,F_{j}}v_{2F_{j}}-b_{2F_{j}})(n-1)b_{2F_{j}}{}^{n-2}$$ (5)

Utility maximization makes the first partial derivative zero to obtain the following:

$$b_{2F_{j}}=\frac{n-1}{n}r_{L,F_{j}}v_{2F_{j}}$$ (6)

Thus, we have shown above that *b*_2*i*_ = *b*(*v*_2*i*_) is only necessary to obtain the maximum value of utility. Now, we prove that we have sufficient conditions. According to Eqs (3) and (6), we get:

$$v_{2L}-b_{2L}=\frac{v_{2L}}{n}$$ (7)

$$r_{L,F_{j}}v_{2F_{j}}-b_{2F_{j}}=\frac{r_{L,F_{j}}v_{2L}}{n}$$ (8)

Putting Eqs (7) and (8) into the right-hand side of Eqs (2) and (5), and rearranging, we get:

$$\frac{\partial U_{2L}}{\partial b_{2L}}=(\frac{n-1}{n}v_{2L}-b_{2L})b_{2L}^{n-2}$$ (9)

$$\frac{\partial U_{2F_{j}}}{\partial b_{2F_{j}}}=(\frac{n-1}{n}r_{L,F_{j}}v_{2F_{j}}-b_{2F_{j}})b_{2F_{j}}{}^{n-2}$$ (10)

When $b_{2L}<\frac{n-1}{n}v_{2L}$, $\frac{\partial U_{2L}}{\partial b_{2L}}>0$. That is $b_{2L}>\frac{n-1}{n}v_{2L}$, $\frac{\partial U_{2L}}{\partial b_{2L}}<0$. Thus, $b_{2L}=\frac{n-1}{n}v_{2L}$ is a sufficient condition for the first stage winner to get the maximum. Similarly, $b_{2F_{j}}=\frac{n-1}{n}r_{L,F_{j}}v_{2F_{j}}$ is a sufficient condition for the first stage loser to obtain a maximum. In summary, the second stage bidding strategy of developer *L* who won the construction land quota in the first stage is $\beta_{2L}^{I}(v_{L})=\frac{n-1}{n}v_{2L}$. The bidding strategy in the second stage of developer *F*_*j*_, who did not win the construction land quota in the first stage, is $\beta_{2F_{j}}^{I}(v_{2F_{j}})=\frac{n-1}{n}r_{L,F_{j}}v_{2F_{j}}$.

In the first stage auction, the expected surplus in advance of the second stage auction depends on the result of the first stage auction. At this point, it is 1. That is, the payment value of the developer who has obtained the construction land quota in the first stage includes not only the actual value of the quota but also the selection value. The latter is reflected in the difference between the expected residual before the auction in the second stage between the developer who has and has not obtained the quota. Therefore, the bidding strategy of the first stage developer is as follows:

$$\beta_{1i}^{I}(v_{i})=\frac{n-1}{n}v_{1i}+\Delta^{I}(r_{L,F_{j}})$$ (11)

As *v*_2*i*_∈[0,1], the expected utility in advance of developer *L*, who won the construction land in the first stage, in the second stage auction is as follows:

$$\begin{array}{l} E[U_{2L}^{I}(v_{2i})]=\iint_{\beta_{2L}>\beta_{2F}}\left(v_{L}-\beta_{2L}\right)(n-1)v_{{}_{2F}}^{n-2}dv_{2L}dv_{2F}\\ \;=\iint_{\beta_{2L}>\beta_{2F}}\left(v_{2L}-\beta_{2L}\right)(n-1)v_{2F}{}^{n-2}dv_{2L}dv_{2F}\\ \;=\iint_{v_{2L}>r_{L,F_{j}}v_{2F}}\left(v_{2L}-\frac{n-1}{n}v_{2L}\right)(n-1)v_{2F}{}^{n-2}dv_{2L}dv_{2F}\\ \;=\int_{0}^{r_{L,F_{j}}}dv_{2L}\int_{0}^{\frac{v_{2L}}{r_{L,F_{j}}}}\frac{1}{n}v_{2L}(n-1)v_{2F}{}^{n-2}dv_{2F}+\int_{r_{L,F_{j}}}^{1}dv_{2L}\int_{0}^{1}\frac{1}{n}v_{2L}(n-1)v_{2F}{}^{n-2}dv_{2F}\\ \;=\frac{1}{n(n+1)}r_{L,F_{j}}{}^{2}+\frac{1}{2n}(1-r_{L,F_{j}}{}^{2}) \end{array}$$

The developer *F* without selection can encounter the following two situations in the bidding of the construction land in the second stage: First, the developer without selection bids higher than the highest bid (the second highest) and the developer with selection bids (the third highest) the highest of the other *n*−2 developers without selection. Second, the developer with no selection outbids the developer with the selection (the second highest) and the highest bid of the other *n*−2 developers with no selection (the third highest). Let $v_{2F_{2}}$ and $\beta_{2F_{2}}$ represent the maximum valuation and maximum equilibrium quotation among *n*−2 developers without selection, respectively, except developer *F* who loses the construction land auction in the second stage. Thus, the expected utility in advance of developer *F*, who did not win in the first stage, for winning the construction land auction in the second stage can be obtained as follows:

$$\begin{array}{l} E[U_{2F}^{I}(v_{2i})]=\underset{\beta_{2F}>\max\{\beta_{2L},\beta_{2F_{2}}\}}{∭}\left(r_{L,F_{j}}v_{2F}-\beta_{2F}\right)(n-2)v_{2F}{}^{n-3}dv_{2L}dv_{2F}dv_{2F_{2}}\\ \;=\underset{rv_{2F}>\max\{v_{2L},r_{L,F_{j}}v_{2F_{2}}\}}{∭}\left(r_{L,F_{j}}v_{2F}-\frac{n-1}{n}r_{L,F_{j}}v_{2F}\right)(n-2)v_{2F}{}^{n-3}dv_{2L}dv_{2F}dv_{2F_{2}}\\ \;=\int_{0}^{r_{L,F_{j}}}dv_{2L}\int_{\frac{v_{L}}{r_{L,F_{j}}}}^{1}dv_{2F}\int_{0}^{\frac{v_{L}}{r_{L,F_{j}}}}\frac{1}{n}r_{L,F_{j}}v_{2F}(n-2)v_{2F}{}^{n-3}dv_{2F_{2}}\\ \;+\int_{0}^{1}dv_{2F_{2}}\int_{v_{2F_{2}}}^{1}dv_{2F}\int_{0}^{r_{L,F_{j}}v_{2F_{2}}}\frac{1}{n}r_{L,F_{j}}v_{2F}(n-2)v_{2F}{}^{n-3}dv_{2L}\\ \;=\frac{1}{n(n+1)}r_{L,F_{j}}{}^{2} \end{array}$$

Thus, according to $\Delta^{I}(r_{L,F_{j}})=E[U_{2L}^{I}(v_{2i})]-E[U_{2F}^{I}(v_{2i})]$, we can calculate that:

$$\Delta^{I}(r_{L,F_{j}})=\frac{1}{2n}(1-r_{L,F_{j}}{}^{2})$$ (12)

When $r_{L,F_{j}}=1$, we have $\Delta^{I}(r_{L,F_{j}})=0$.

Q.E.D.

### Equilibrium bidding price of the second-price sealed-bid sequential auction

The reverse recursion method is used to solve the developers’ balanced quotation strategy for construction land quotas and construction land in second-price sealed-bid sequential auction. Subsequently, the following proposition 2 is obtained:

#### Proposition 2

For second-price sealed-bid sequential auction format, the developers’ equilibrium bidding strategies in the two stages are as follows:

$$\beta_{{}_{1i}}^{II}(v_{i})=v_{1i}+\Delta^{II}(r_{L,F_{j}})$$

$$\left\{ \begin{array}{l} \beta_{{}_{2L}}^{II}(v_{2L})=v_{2L}\\ \beta_{{}_{2F_{j}}}^{II}(v_{2j})=r_{L,F_{j}}v_{2F_{j}},j=1,2,\ldots ,n-1 \end{array}\right.$$

where, $\Delta^{II}(r_{L,F_{j}})=\frac{n-2}{2n}r_{L,F_{j}}{}^{2}-\frac{n-1}{n}r_{L,F_{j}}+\frac{1}{2}$, $0\leq r_{L,F_{j}}\leq 1$.

Proof: Consider the second stage of the auction. Consistent with the first-price sequential auction solution method, the utility function of winner *L* in the second-price sequential auction can be obtained as $U_{L}=\int_{0}^{1}\left(v_{2L}-b_{2L}\right)d{\left(b_{2L}\right)}^{n-1}$. Then, we derive the partial derivative of the utility function to obtain the first-order condition:

$$\frac{\partial U_{2L}}{\partial b_{2L}}=(v_{2L}-b_{2L})(n-1)b_{2L}^{n-2}$$ (13)

Utility maximization makes the first partial derivative zero to obtain the following:

$$b_{2L}=v_{2L}$$ (14)

Similarly, the utility function of loser *F*_*j*_ is $U_{F_{j}}=\int_{0}^{1}\left(r_{L,F_{j}}v_{2F_{j}}-b_{2F_{j}}\right)d{\left(b_{2F_{j}}\right)}^{n-1},j=1,2,\ldots ,n-1$. The utility is maximized by setting the first partial derivative to zero and we obtain:

$$b_{2F_{j}}=r_{L,F_{j}}v_{2F_{j}}$$ (15)

Similar to the first-price sealed-bid sequential auction proof, we easily prove that it satisfies sufficient conditions. In conclusion, in the second stage, the bidding strategy of the developer who won the construction land quota in the first stage is *β*_2*L*_ = *v*_*L*_. Correspondingly, developers who did not win the construction land quota in the first stage had a bidding strategy $\beta_{2F_{j}}=r_{L,F_{j}}v_{2F_{j}}$.

Now consider the first stage strategy. Each bidder’s expected surplus in the second stage auction depends on the outcome of the first stage. Therefore, the value of the bidder in the first stage auction is the value of the first item plus parameter $\Delta^{II}(r_{L,F_{j}})$. This represents the value of the selection that can be obtained by entering the second stage auction as the winner compared with the loser in the first stage; that is, the difference of the pre-expected residual $\Delta^{II}(r_{L,F_{j}})=\Delta E(U_{i})$. Since $\Delta^{II}(r_{L,F_{j}})$ is the same for both bidders in the first-stage auction, the first-stage auction is symmetric. Therefore, we can easily prove that the Nash equilibrium strategy in the first-stage auction is:

$$\beta_{1i}(v_{1i})=v_{1i}+\Delta^{II}(r_{L,F_{j}})$$ (16)

According to *v*_2*i*_∈[0,1], the expected utility in advance of the winning developer *L* in the first stage to continue in the construction land auction in the second stage can be calculated as follows:

$$\begin{array}{l} E[U_{2L}^{II}(v_{i})]=\iint_{\beta_{2L}\geq \beta_{2F}}\left(v_{2L}-\beta_{2F}\right)(n-1)v_{2F}{}^{n-2}dv_{2L}dv_{2F}\\ \;=\iint_{v_{2L}\geq r_{L,F_{j}}v_{2F}}\left(v_{2L}-r_{L,F_{j}}v_{2F}\right)(n-1)v_{2F}{}^{n-2}dv_{2L}dv_{2F}\\ \;=\int_{0}^{r_{L,F_{j}}}dv_{2L}\int_{0}^{\frac{v_{2L}}{r_{L,F_{j}}}}(v_{2L}-r_{L,F_{j}}v_{2F})(n-1)v_{2F}{}^{n-2}dv_{2F}+\int_{r_{L,F_{j}}}^{1}dv_{2L}\int_{0}^{1}(v_{2L}-r_{L,F_{j}}v_{2F})(n-1)v_{2F}{}^{n-2}dv_{2F}\\ \;=\frac{n-1}{2(n+1)}r_{L,F_{j}}{}^{2}-\frac{n-1}{n}r_{L,F_{j}}+\frac{1}{2} \end{array}$$

There are two situations in which the developer *F*, who has no selection, auctions the construction land in the second stage, which is similar to the first-price sealed-bid sequential auction. In the second stage, the expected utility of the developers who did not win the first stage construction land auction is as follows:

$$\begin{array}{l} E[U_{2F}^{II}(v_{i})]=\underset{\beta_{2F}>\max\{\beta_{2L},\beta_{2F_{2}}\}}{∭}\left(\beta_{2F}-\max\{\beta_{2L},\beta_{2F_{2}}\}\right)(n-2)v_{2F_{2}}{}^{n-3}dv_{2L}dv_{2F}dv_{2F_{2}}\\ \;=\underset{r_{L,F_{j}}v_{2F}>\max\{v_{2L},r_{L,F_{j}}v_{2F_{2}}\}}{∭}\left(r_{L,F_{j}}v_{2F}-\max\{v_{2L},r_{L,F_{j}}v_{2F_{2}}\}\right)(n-2)v_{2F_{2}}{}^{n-3}dv_{2L}dv_{2F}dv_{2F_{2}}\\ \;=\int_{0}^{r_{L,F_{j}}}dv_{2L}\int_{\frac{v_{2L}}{r_{L,F_{j}}}}^{1}dv_{2F}\int_{0}^{\frac{v_{2L}}{r_{L,F_{j}}}}(r_{L,F_{j}}v_{2F}-v_{2L})(n-2)v_{2F_{2}}{}^{n-3}dv_{2F_{2}}\\ \;+\int_{0}^{1}dv_{2F_{2}}\int_{v_{2F_{2}}}^{1}dv_{2F}\int_{0}^{r_{L,F_{j}}v_{2F_{2}}}(r_{L,F_{j}}v_{2F}-rv_{2F_{2}})(n-2)v_{2F_{2}}{}^{n-3}dv_{2L}\\ \;=\frac{1}{n(n+1)}r_{L,F_{j}}{}^{2} \end{array}$$

According to $\Delta^{II}(r_{L,F_{j}})=E[U_{2L}^{II}(v_{i})]-E[U_{2F}^{II}(v_{i})]$, we get:

$$\Delta^{II}(r_{L,F_{j}})=\frac{n-2}{2n}r_{L,F_{j}}{}^{2}-\frac{n-1}{n}r_{L,F_{j}}+\frac{1}{2}$$ (17)

When $r_{L,F_{j}}=1$, we have $\Delta^{II}(r_{L,F_{j}})=0$.

Q.E.D.

### Farmers’ expected income

#### Proposition 3

For the first- and second-price sealed-bid sequential auction format, farmers’ expected income is:

$$E[R^{I}]=\frac{n-1}{n+1}\varepsilon^{n+1}+\Delta^{I}(r_{L,F_{j}})\varepsilon^{n}$$

$$E[R^{II}]=\frac{n-1}{n+1}\varepsilon^{n+1}+\Delta^{II}(r_{L,F_{j}})\varepsilon^{n}$$

Proof: In a first-price sealed-bid sequential auction, the winner pays his or her bid. Therefore, the expected payoff for the bidder with the value of *v*_1*i*_ in the first stage of paying *m*(*v*_1*i*_) is $E[m(v_{1i})]=\int_{0}^{\varepsilon }m(v_{1i})(n-1)f^{n-2}(v_{1i})dv_{1i}$. There are three ways in which developers win, as shown in Table 2:

**Table 2:** Developers winning and paying prices for the first-price format.

Wins for developers	Stage 1	Stage 1 Payment
construction land quota and construction land	*β*_1*L*_>*β*_1*F*_	$\frac{n-1}{n}v_{1L}+\Delta^{I}(r_{L,F_{j}})$
construction land quota	*β*_1*L*_>*β*_1*F*_	$\frac{n-1}{n}v_{1L}+\Delta^{I}(r_{L,F_{j}})$
construction land	*β*_1*L*_<*β*_1*F*_	0

From Table 2, we find that the developer wins the auction in three cases: First, when developer *i* obtains both the construction land quota and the construction land, the winning developer’s quotation in the first stage is greater than the maximum quotation of *n*−1 losing developers. Furthermore, the winning developer’s quotation in the second stage is also greater than the maximum quotation of *n*−1 losing developers. In this case, the price paid by the winning developer for the construction land quota is the quotation. Second, when developer *i* only obtains the quota of construction land, the winning developer’s quotation in the first stage is greater than the maximum quotation of *n*−1 losers. Furthermore, the winning developer’s quotation in the second stage is less than the maximum quotation of *n*−1 losers. In this case, the price paid by the winning developer for the quota of construction land is the quotation. Third, when developer *i* only obtains construction land, the winning developer’s quotation in the first stage is less than the maximum quotation of *n*−1 losers. Furthermore, the maximum quotation of the losing developer in the second stage is greater than the winning developer and other *n*−2 losing developers. In this case, the price paid by the developer for construction land is zero. Therefore, the expected income of the quota auction of construction land in the first stage can be calculated. That is, farmers’ expected income *E*[*R*^*I*^] (the superscript I represents the first-price sealed-bid sequential auction) is the sum of the expected payment in advance of all bidders in the first stage. Thus:

$$\begin{array}{l} E[R^{I}]=n\int_{0}^{\varepsilon }dv_{1L}\int_{0}^{v_{1L}}\left(n-1\right)v_{1F}^{n-2}dv_{1F}\int_{0}^{r_{L,F_{j}}}dv_{2L}\int_{0}^{\frac{v_{2L}}{r_{L,F_{j}}}}\left(\frac{n-1}{n}v_{1L}+\Delta^{I}(r_{L,F_{j}})\right)(n-1)v_{2F}^{n-2}dv_{2F}\\ \;+n\int_{0}^{\varepsilon }dv_{1L}\int_{0}^{v_{1L}}\left(n-1\right)v_{1F}^{n-2}dv_{1F}\int_{r}^{1}dv_{2L}\int_{0}^{1}\left(\frac{n-1}{n}v_{1L}+\Delta^{I}(r_{L,F_{j}})\right)(n-1)v_{2F}^{n-2}dv_{2F}\\ \;+n\int_{0}^{\varepsilon }dv_{1L}\int_{0}^{v_{1L}}\left(n-1\right)v_{1F}^{n-2}dv_{1F}\int_{0}^{r_{L,F_{j}}}dv_{2L}\int_{\frac{v_{2L}}{r_{L,F_{j}}}}^{1}\left(\frac{n-1}{n}v_{1L}+\Delta^{I}(r_{L,F_{j}})\right)(n-1)v_{2F}^{n-2}dv_{2F}\\ \;=\frac{n-1}{n+1}\varepsilon^{n+1}+\Delta^{I}(r_{L,F_{j}})\varepsilon^{n} \end{array}$$ (18)

Similarly, in sequential bivalent auction, there are three winning situations for developers, as shown in Table 3:

**Table 3:** Developers winning and paying prices for the second-price format.

Wins for developers	Stage 1	Stage 1 Payment
Construction land quota and construction land	*β*_1*L*_>*β*_1*F*_	$v_{1F}+\Delta^{II}(r_{L,F_{j}})$
Construction land quota	*β*_1*L*_>*β*_1*F*_	$v_{1F}+\Delta^{II}(r_{L,F_{j}})$
Construction land	*β*_1*L*_<*β*_1*F*_	0

From Table 3, we find that there are also three situations in which the developer auctions: 1) both the construction land quota and the construction land, 2) only the construction land quota and the construction land quota, and 3) only the construction land. For the specific situation, refer to the analysis in Table 2 of the first-price sealed-bid sequential auction. Therefore, the expected income obtained from the quota auction of construction land in the first stage can be calculated, that is, farmers’ expected income under the second sequential auction of price. The expected return *E*[*R*^*II*^] (superscript II represents the auction of the second-price sealed-bid sequential auction) of the construction land obtained by farmers is the sum of the expected payment in advance of all bidders in the first stage. Thus:

$$\begin{array}{l} E[R^{II}]=n\int_{0}^{\varepsilon }dv_{1L}\int_{0}^{v_{1L}}\left(n-1\right)v_{1F}^{n-2}dv_{1F}\int_{0}^{r_{L,F_{j}}}dv_{2L}\int_{0}^{\frac{v_{2L}}{r_{L,F_{j}}}}\left(v_{1F}+\Delta^{II}(r_{L,F_{j}})\right)(n-1)v_{2F}^{n-2}dv_{2F}\\ \;+n\int_{0}^{\varepsilon }dv_{1L}\int_{0}^{v_{1L}}\left(n-1\right)v_{1F}^{n-2}dv_{1F}\int_{r}^{1}dv_{2L}\int_{0}^{1}\left(v_{1F}+\Delta^{II}(r_{L,F_{j}})\right)(n-1)v_{2F}^{n-2}dv_{2F}\\ \;+n\int_{0}^{\varepsilon }dv_{1L}\int_{0}^{v_{1L}}\left(n-1\right)v_{1F}^{n-2}dv_{1F}\int_{0}^{r_{L,F_{j}}}dv_{2L}\int_{\frac{v_{2L}}{r_{L,F_{j}}}}^{1}\left(v_{1F}+\Delta^{II}(r_{L,F_{j}})\right)(n-1)v_{2F}^{n-2}dv_{2F}\\ \;=\frac{n-1}{n+1}\varepsilon^{n+1}+\Delta^{II}(r_{L,F_{j}})\varepsilon^{n} \end{array}$$ (19)

Q.E.D.

### Equilibrium solution analysis

The equilibrium solution of construction land quota and the farmers’ expected income under the first- and second-price sealed-bid sequential auctions is summarized in Table 4:

**Table 4:** Developers’ equilibrium bidding price and farmers’ expected income.

Auction type		First-stage equilibrium bidding price	Second-stage equilibrium bidding price	Expected income
First-price sealed-bid sequential auction	Quality selection	$\beta_{1i}^{I}(v_{1i})=\frac{n-1}{n}v_{1i}+\Delta^{I}(r_{L,F_{j}})$ $\Delta^{I}(r_{L,F_{j}})=\frac{1}{2n}(1-r_{L,F_{j}}{}^{2})$	$\left\{ \begin{array}{l} \beta_{{}_{2L}}^{I}(v_{2L})=\frac{n-1}{n}v_{2L}\\ \beta_{{}_{2F_{j}}}^{I}(v_{2j})=\frac{n-1}{n}r_{L,F_{j}}v_{2F_{j}} \end{array}\right.$	$\frac{n-1}{n+1}\varepsilon^{n+1}+\Delta^{I}(r_{L,F_{j}})\varepsilon^{n}$
	No quality selection	$\beta_{{}_{1}}^{I}(v_{1i})=\frac{n-1}{n}v_{1i}$ $\Delta^{I}(r_{L,F_{j}})=0$	$\beta_{{}_{2i}}^{I}(v_{2i})=\frac{n-1}{n}v_{2i}$	$\frac{n-1}{n+1}\varepsilon^{n+1}$
Second-price sealed-bid sequential auction	Quality selection	$\beta_{{}_{1i}}^{II}(v_{1i})=v_{1i}+\Delta^{II}(r_{L,F_{j}})$ $\Delta^{II}(r)=\frac{n-2}{2n}r_{L,F_{j}}{}^{2}-\frac{n-1}{n}r_{L,F_{j}}+\frac{1}{2}$	$\left\{ \begin{array}{l} \beta_{{}_{2L}}^{II}(v_{2L})=v_{2L}\\ \beta_{{}_{2F_{j}}}^{II}(v_{2j})=r_{L,F_{j}}v_{2F_{j}} \end{array}\right.$	$\frac{n-1}{n+1}\varepsilon^{n+1}+\Delta^{II}(r_{L,F_{j}})\varepsilon^{n}$
	No quality selection	$\beta_{{}_{1i}}^{II}(v_{1i})=v_{1i}$ $\Delta^{II}(r_{L,F_{j}})=0$	$\beta_{{}_{2i}}^{II}(v_{2i})=v_{2i}$	$\frac{n-1}{n+1}\varepsilon^{n+1}$
		$\Delta^{II}(r_{L,F_{j}})=0$		

According to Table 4, due to the existence of selection, all developers enter the second-stage construction land auction with and without selection. This induces a difference in expected income, thus improving the independent value of the first-stage construction land quota, namely the selection value. We find that the selection value of construction land quota can improve the quota equilibrium quotation and achieve the purpose of increasing farmers’ expected income. In addition, we find that the number of developers participating in the auction will affect the quota and the balanced quotation of construction land. Numerous studies have also analyzed and discussed the number of developers [38–41]. Therefore, we posit:

#### Proposition 4

The selection improves the developers’ equilibrium bidding price and farmers’ expected income.

Proof: First, we prove that the selection improves the construction land quota balanced quotation. Under the first-price sealed-bid sequential auction, according to Eq (11), the quota equilibrium quotation considering that the condition that there is no selection and when $r_{L,F_{j}}=1$ is:

$$\beta_{{}_{1i}}^{I*}(v_{i})=\frac{n-1}{n}v_{i}$$ (20)

Subtracting Eq (11) from Eq (20), we get $\Delta \beta_{1i}^{I}(v_{i})=\beta_{1i}^{I}(v_{i})-\beta_{1i}^{I*}(v_{i})=\Delta^{I}(r_{L,F_{j}})$.

Because $0\leq r_{L,F_{j}}\leq 1$ and *n*≥2, we get $\Delta^{I}(r_{L,F_{j}})=\frac{1}{2n}(1-r_{L,F_{j}}{}^{2})\geq 0$ according to Eq (12). Similarly, according to Eq (16), under the second-price sealed-bid sequential auction, the equilibrium quotation for the construction land quota when the developer has no selection is $\beta_{{}_{1}}^{II}(v_{i})=v_{i}$. Then, we can obtain $\Delta \beta_{1i}^{II}(v_{i})=\Delta^{II}(r_{L,F_{j}})\geq 0$ by subtraction. Therefore, no matter the first- or the second-price sealed-bid sequential auction, the selection will improve the first-stage developer’s equilibrium bid for the construction land quota.

Next, we show that the selection increases farmers’ expected income. In the case of the first-price sealed-bid sequential auction, in Eq (18), we set $r_{L,F_{j}}=1$ to obtain the farmer’s expected income without selection:

$$E^{*}[R^{I}]=\frac{n-1}{n+1}\varepsilon^{n+1}$$ (21)

Subtracting Eq (21) from Eq (18), we get $\Delta E[R^{I}]=E[R^{I}]-E^{*}[R^{I}]=\frac{1}{2n}(1-r_{L,F_{j}}{}^{2})\varepsilon^{n}>0$. Similarly, if $r_{L,F_{j}}=1$ is used in Eq (19), the expected income of farmers under the second-price sealed-bid sequential auction is $E^{*}[R^{II}]=\frac{n-1}{n+1}\varepsilon^{n+1}$. Then, we can obtain $\Delta E[R^{II}]=(\frac{n-2}{2n}r_{L,F_{j}}{}^{2}-\frac{n-1}{n}r_{L,F_{j}}+\frac{1}{2})\varepsilon^{n}>0$ by subtracting the formula. This proves that the selection increases farmers’ expected income in the case of the second-price sealed-bid sequential auction. Further, when $r_{L,F_{j}}=1$, E*[*R*^*I*^] = E*[*R*^*II*^]. Then, the income equivalence theorem is satisfied. However, when $r_{L,F_{j}}\neq 1$, E*[*R*^*I*^]≠E*[*R*^*II*^]. Then the income equivalence theorem is not valid; that is, the selection of construction land quota leads to the failure of the income equivalence theorem.

*Q*.*E*.*D*. Proposition 4 shows that no matter the first- or second-price sealed-bid sequential auction, the selection can improve the equilibrium price of the developer on the construction land quota and farmers’ expected income. Therefore, developers can achieve the right to choose the expected construction land by obtaining the construction land quota to reduce the degree of matching of the construction land in the second stage for developers who do not have the right to choose. Furthermore, they can obtain the bidding advantage in the second stage. For farmers, the hope is that the government can grant the construction land quota selection to improve their expected income. With regard to the government, it can give the right to use construction land quota selection. Furthermore, in the second stage, it should divide the developers participating in the auction into the selection and no selection classes. Intensify competition between construction land bidding stage developers to improve the equilibrium bidding of construction land quota, raising farmers’ expected return, encouraging the enthusiasm of farmers reclamation, realize the reuse of a large number of idle homesteads in rural areas, and reallocating land resources is of great significance for promoting new urbanization in China.

#### Proposition 5

The greater the value of the selection to the developer, the higher the equilibrium price of the construction land quota and farmers’ expected income.

Proof: First, we prove the relationship between the value of the selection and the equilibrium quotation of the construction land: that is, we prove that the land depreciation coefficient $r_{L,F_{j}}$ is negatively correlated with the equilibrium quotation of the land. In the first- and second-price sealed-bid sequential auctions, the partial derivatives of Eqs (11) and (16) against the land depreciation coefficient $r_{L,F_{j}}$ can are $\frac{\partial \beta_{1i}^{I}}{\partial r_{L,F_{j}}}=\frac{-r_{L,F_{j}}}{n}\leq 0$ and $\frac{\partial \beta_{1i}^{II}}{\partial r_{L,F_{j}}}=\frac{n-2}{n}r_{L,F_{j}}-\frac{n-1}{n}\leq \frac{n-1}{n}(r_{L,F_{j}}-1)\leq 0$, respectively. Therefore, whether it is the first- or the second-price sealed-bid sequential auction, there is a negative correlation between the land devaluation coefficient and the balanced quotation of construction land quota. That is, with a decrease in $r_{L,F_{j}}$, for a greater degree of construction land devaluation for the developers who have no right of selection, they will improve the balanced quotation of the quota to obtain the right of selection of the quota. This is because the smaller $r_{L,F_{j}}$ is, the lower the degree of matching between the construction land auctioned in the second stage and the expected construction land of the developer without selection, and the lower the applicability of the project to be developed by the developer. Therefore, the developer is willing to pay a higher equilibrium price for the quota selection.

Then, we prove the relationship between the value of the selection and the balanced quotation of the construction land quota. That is, we prove that the land depreciation coefficient $r_{L,F_{j}}$ is negatively correlated with farmers’ expected income. According to Eqs (18) and (19), $r_{L,F_{j}}\in [0,1]$, and *n*≥2, we can find that the first order partial derivatives of farmers’ expected income to land depreciation coefficient under the first- and second-price sealed-bid sequential auctions are $\frac{\partial E[R^{I}]}{\partial r_{L,F_{j}}}=-\frac{1}{n}\varepsilon^{n}r_{L,F_{j}}\leq 0$ and $\frac{\partial E[R^{II}]}{\partial r_{L,F_{j}}}=(\frac{n-2}{n}r_{L,F_{j}}-\frac{n-1}{n})\varepsilon^{n}\leq \frac{n-1}{n+1}(r_{L,F_{j}}-1)\varepsilon^{n}\leq 0$, respectively. Thus, we prove that whether it is the first- or second-price sealed-bid sequential auction, the greater the value of the selection to the developer, the higher the farmers’ expected income.

*Q*.*E*.*D*. Proposition 5 shows that the greater the value of the selection to the developer–that is, the smaller the land depreciation coefficient–farmers can get higher expected income. A smaller coefficient means that the degree of matching between the construction land and the land expected by the developer without the selection is lower, and the degree of depreciation of the selected construction land to the developer without the selection is higher. Therefore, for the developers who have the right to choose, the selected construction land is the expected construction land of the proposed development project. This increases the degree of depreciation of the construction land to the developers who have no right to choose and improves their bidding advantages. For farmers, the higher the balanced price of construction land, the higher the expected income. For the government, it is necessary to expand the scope of alternative construction land to increase the matching difference between construction land and developers, thereby further intensifying the competition among developers. This will help improve the balanced quotation of construction land quota, increase farmers’ income, effectively dispose of and make full use of idle land, standardize land market behavior, and promote the purpose of saving and intensive land use.

#### Proposition 6

The developer’s equilibrium bidding price and farmers’ expected income are always higher in the second-price sealed-bid sequential auction than in the first-price sealed-bid sequential auction.

Proof: First, we prove that the equilibrium quotation of the construction land quota is always higher in the second-price sealed-bid sequential auction than that under the first-price sealed-bid sequential auction. Let $\Delta^{*}\beta_{1i}=\beta_{{}_{1i}}^{I}-\beta_{{}_{1i}}^{II}$ denote the difference between the developer’s equilibrium quotations for construction land quotas under these two types of auctions. From Eqs (11) and (16), we can show that Δ*β_1*i*_<0. That is, the developer’s equilibrium price for construction land quotas is lower under the first-price sealed-bid sequential auction than that under the second-price sealed-bid sequential auction.

Next, we prove that farmers’ expected income is always higher under the second-price sealed-bid sequential auction than under the first-price sealed-bid sequential auction. Let Δ*E[*R*] = *E*[*R*^*I*^]−*E*[*R*^*II*^] denote the difference between the farmers’ expected income under these two types of auctions. From Eqs (18) and (19), we can show that Δ*E[*R*]≤0. That is, farmers’ expected income is higher under the second-price sealed-bid sequential auction than under the first-price sealed-bid sequential auction.

*Q*.*E*.*D*. Proposition 6 shows that the developer’s equilibrium quotation for construction land quotas and the farmers’ expected income are always higher under the second-price sealed-bid sequential auction than in the first-price one. Therefore, for farmers, the hope is that the government will auction construction land quotas and construction land through the second-price sealed-bid sequential auction, to obtain higher expected returns. For the government, providing the function of selecting the right of construction land quota and choosing the second-price sealed-bid sequential auction can increase the equilibrium bidding price of the construction land quota and farmers’ expected income. The allocation of land provides a theoretical basis for promoting the overall development of China’s urban and rural areas, optimizing the structure of urban and rural land use, and improving the rural land transfer system.

#### Proposition 7

The number of developers is negatively correlated with the equilibrium bidding price of construction land quotas and farmers’ expected income.

Proof: First, we prove the negative correlation between the number of developers and the equilibrium quotation of construction land quotas. Under the first-price sealed-bid sequential auction, according to Eqs (11) and (12), the first-order partial derivative of the equilibrium quotation of construction land quota with respect to the number of developers is $\frac{\partial \beta_{1i}^{I}(v_{i})}{\partial n}=\frac{v_{i}-n(1-r_{L,F_{j}}{}^{2})}{n^{2}}\leq \frac{v_{i}-n}{n^{2}}\leq 0$. Similarly, according to Eqs (16) and (17), the first-order partial derivative of the equilibrium quotation under the second-price sealed-bid sequential auction with respect to the number of developers participating in the auction can be obtained as $\frac{\partial \beta_{1i}^{II}(v_{i})}{\partial n}=\frac{(n-2)r_{L,F_{j}}}{n}-\frac{n-1}{n}\leq \frac{n-1}{n}(r_{L,F_{j}}-1)\leq 0$. Thus, under both auctions, the number of developers is negatively correlated with the equilibrium quotation of the quota.

Then, we prove the negative correlation between the number of developers and farmers’ expected income. According to Eq (18), the first-order partial derivative of the expected income of farmers with respect to the number of developers under the first-price sealed-bid sequential auction is:

$$\begin{array}{l} \frac{\partial E[R^{I}]}{\partial n}=\frac{2}{{\left(n+1\right)}^{2}}\varepsilon^{n+1}+\frac{n-1}{n+1}\varepsilon^{n+1}\ln\varepsilon -\frac{\left(1-r_{L,F_{j}}{}^{2}\right)}{2n^{2}}\varepsilon^{n}+\frac{\left(1-r_{L,F_{j}}{}^{2}\right)}{2n}\varepsilon^{n}\ln\varepsilon \\ \;=\frac{\varepsilon^{n+1}}{{\left(n+1\right)}^{2}}[2+(n^{2}-1)\ln\varepsilon ]+\frac{\left(1-r_{L,F_{j}}{}^{2}\right)}{2n^{2}}\varepsilon^{n}(n\ln\varepsilon -1) \end{array}$$ (22)

Let $X=\frac{\varepsilon^{n+1}}{{\left(n+1\right)}^{2}}[2+(n^{2}-1)\ln\varepsilon ]$ and $Y=\frac{\left(1-r_{L,F_{j}}{}^{2}\right)}{2n^{2}}\varepsilon^{n}(n\ln\varepsilon -1)$. When *X* = 0, $\varepsilon =e^{-\frac{2}{3}}=\frac{1}{\sqrt[{3}]{e^{2}}}\approx 0.6$. When *Y* = 0, $\varepsilon =e^{\frac{1}{2}}=\sqrt{e}\approx 1.6$. According to data from the Chongqing Land Exchange, the quota transaction price of construction land is much smaller than the transaction price of construction land at approximately 0.1 times the price of the land. Therefore, $\varepsilon <e^{-\frac{2}{3}}<e^{\frac{1}{2}}$. Thus, $\frac{\partial E[R^{II}]}{\partial n}\leq 0$. That is, under the condition of the selection of construction land quota, whether under the first- or the second-price sealed-bid sequential auctions, the number of developers participating in the auction is negatively correlated with the equilibrium quotation of the construction land quota.

*Q*.*E*.*D*. Proposition 7 shows that regardless of whether the first- or second-price sealed-bid sequentially auction occurs, the quota equilibrium quotation and the number of developers are negatively correlated. Therefore, the government grants the right to choose construction land quotas. At the same time, by reasonably controlling and improving the structure of the number of developers participating in the auction, the balanced quotation of the construction land quotas can be increased, and farmers’ income and enthusiasm for reclamation can be improved. The problem of a large number of idle homesteads in the rural areas of our country today ensures the effective use of rural land resources and promotes the scientific development of urban and rural areas.

## Numerical analyses

### Bidder’s transaction price

Assuming that each bidder’s valuation of construction land is a random number, using Matlab to simulate 10,000 auctions, we get: Under the first-price (second-price) sealed-bid sequential auction with selection, the developer with the highest bid wins the quota and the transaction bidding price is the highest price (second highest quotation). Based on the practice and the model, it is assumed that all developers’ estimates of the quota and construction land are random numbers of [0.0.1] and [0,1] respectively. Meanwhile, the equilibrium quotation can be obtained by solving (Tables 5 and 6).

**Table 5:** Transaction bidding price of bidders for the first-price format.

Bidder		2	3	4	5	6	7	8	9	10	11	12
Value	*v*_1*i*_	0.089	0.030	0.095	0.054	0.073	0.097	0.033	0.012	0.091	0.062	0.028
	*v*_2*i*_	0.841	0.486	0.389	0.235	0.094	0.972	0.937	0.804	0.021	0.481	0.919
Price	$\beta_{1i}^{I}$	0.232	0.145	0.165	0.118	0.123	0.136	0.075	0.052	0.120	0.090	0.057
	$\beta_{2i}^{I}$	0.420	0.324	0.292	0.188	0.078	0.833	0.820	0.715	0.019	0.438	0.842

**Table 6:** Transaction bidding price of bidders for the second-price format.

Bidder		2	3	4	5	6	7	8	9	10	11	12
Value	*v*_1*i*_	0.047	0.090	0.047	0.052	0.018	0.065	0.035	0.069	0.062	0.074	0.025
	*v*_2*i*_	0.105	0.763	0.921	0.029	0.675	0.548	0.034	0.576	0.097	0.764	0.475
Price	$\beta_{1i}^{II}$	0.297	0.299	0.234	0.227	0.185	0.226	0.192	0.222	0.212	0.221	0.170
	$\beta_{2i}^{II}$	0.052	0.381	0.460	0.014	0.337	0.274	0.017	0.288	0.048	0.382	0.238

**Fig 1:**
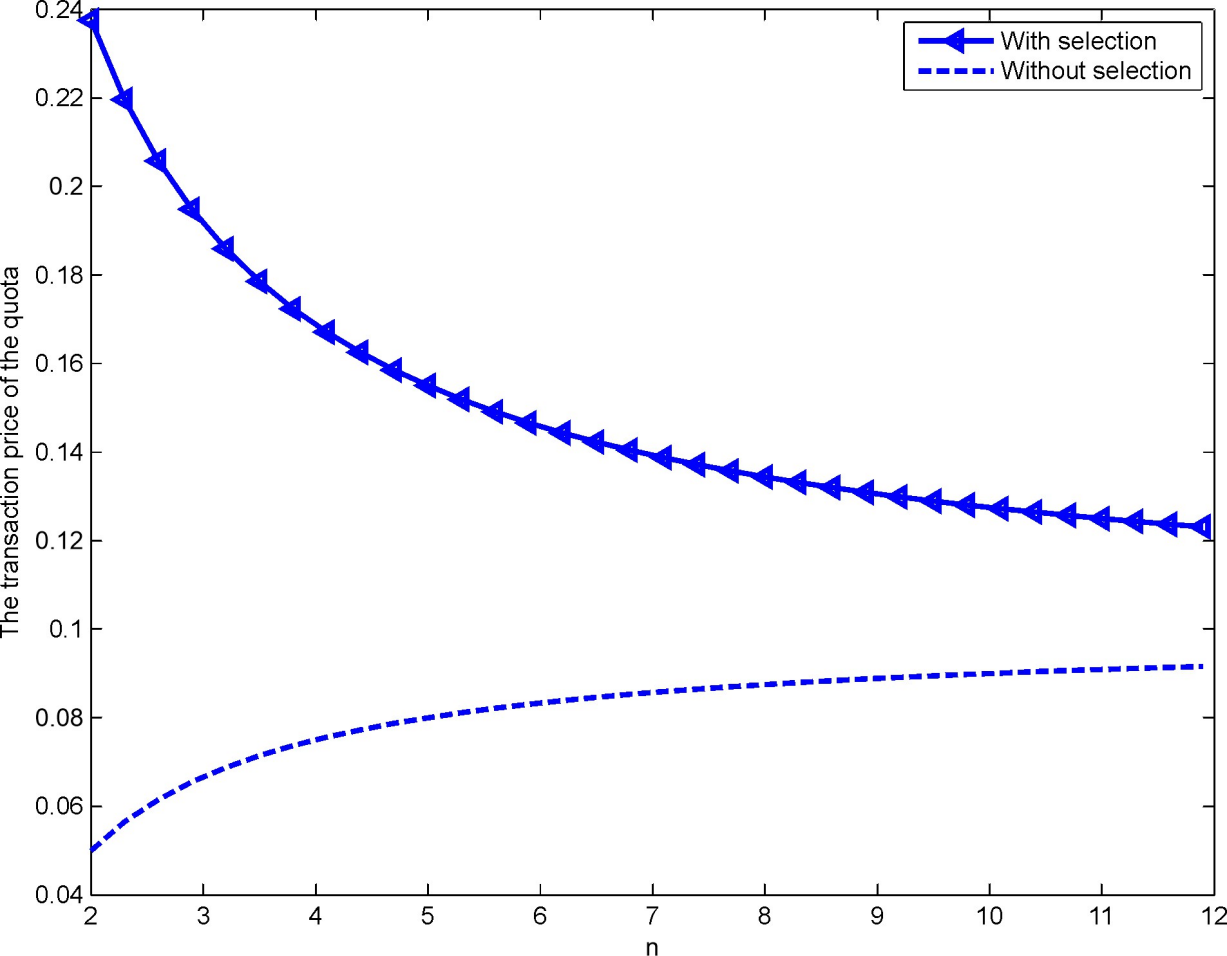
Impact of *n* on the transaction price of the quota for the first-price format.

**Fig 2:**
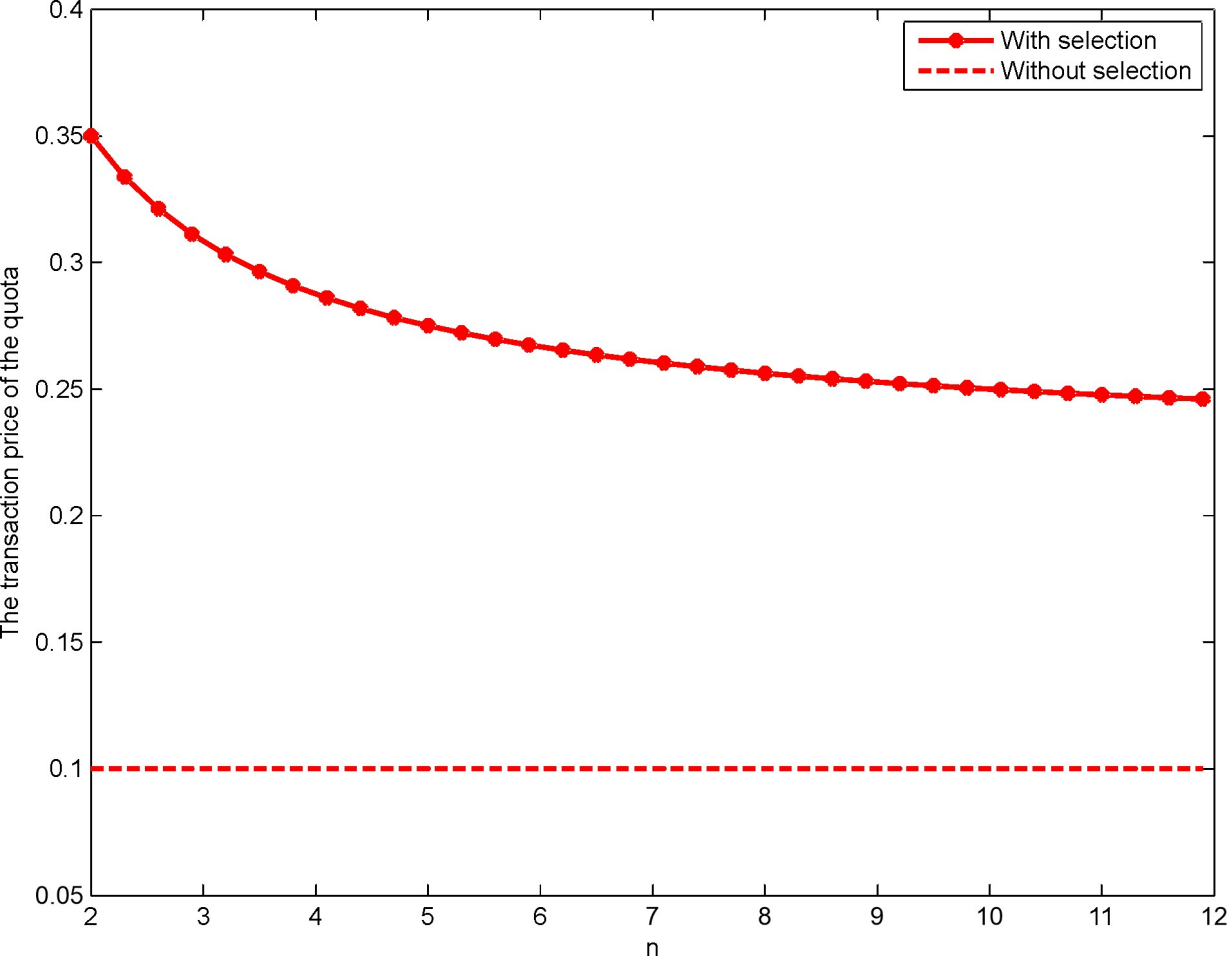
Impact of *n* on the transaction price of the quota for the second-price format.

This study is based on Maskin and Riley’s [42] method. Let *n*∈[2,12]. Then, we carry out a numerical analysis of the transaction prices of construction land quotas in the first- and the second-price sealed-bid sequential auction as shown in Figs 1 and 2, respectively:

The triangle line and the star line in Figs 1 and 2, respectively, represent the developer’s transaction price for the construction land quota when there is an selection under the first- and the second-price sealed-bid sequential auctions, respectively. The dotted line represents the developer’s price for construction land quota when there is no selection. Notably, whether it is a first- or a second-price sealed-bid sequential auction, the developer’s transaction price for construction land quotas with the selection is significantly higher than without the selection, thus demonstrating Proposition 4. In addition, there is a negative correlation between the number of developers and the transaction price of the quotas when there is a right to choose. This verifies the research conclusions of Sørensen [20], Wang and Liu [39], Zhang and Wang [40], and Jing and Li [41], among others, and demonstrates Proposition 7. Therefore, to achieve the goal of reducing and reusing a large number of idle homesteads in rural areas, the government increases the transaction price of the quotas by granting the right to choose the quotas while reasonably controlling the number of developers participating in the auction of the quotas.

**Fig 3:**
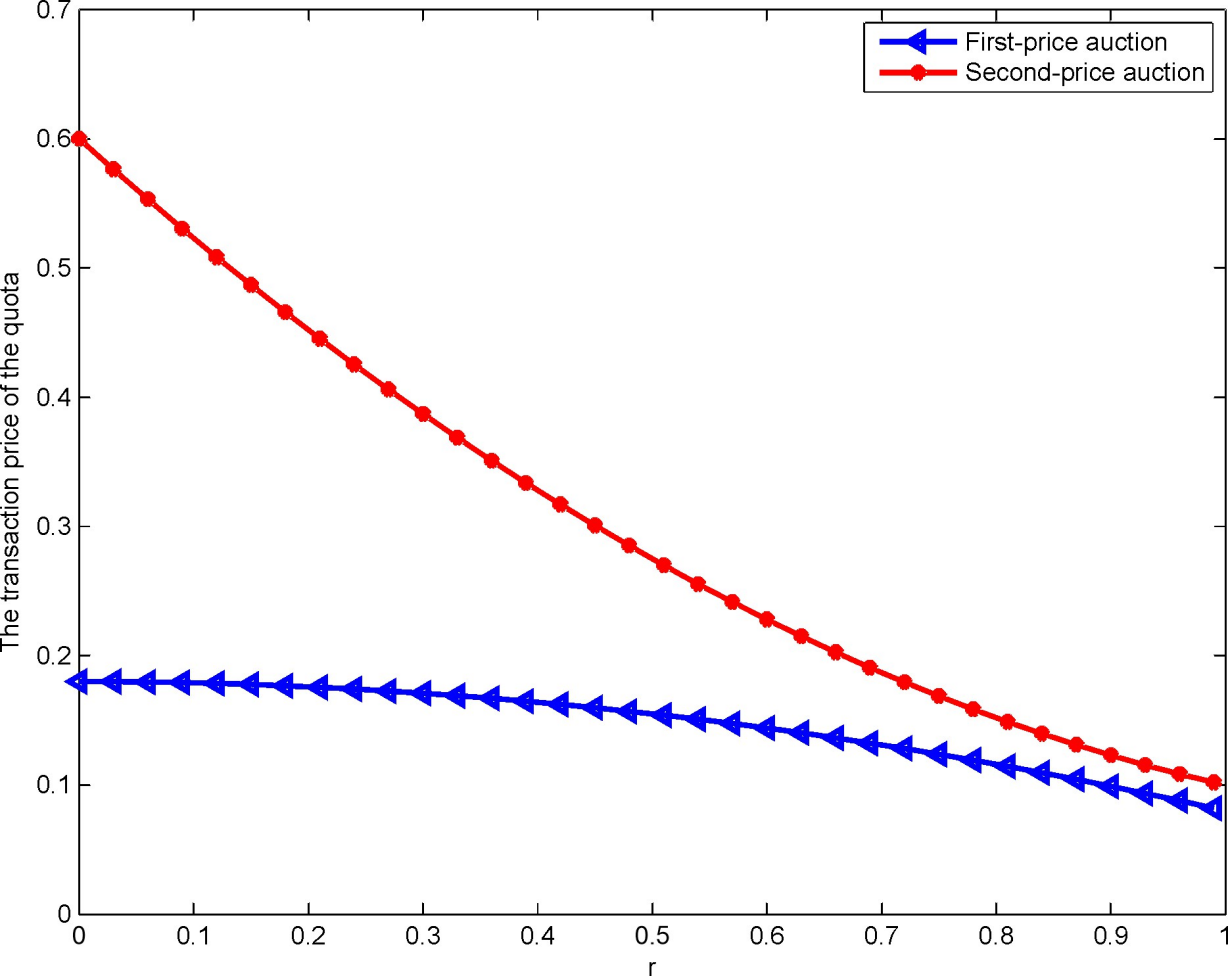
Impact of $r_{L,F_{j}}$ on the transaction price of the quota.

Fig 3 show whether the first- or the second-price sealed-bid sequential auction is better for construction land quota transaction prices when there is a right of selection:

Notably, for both auctions, the land depreciation coefficient and the transaction price are negatively correlated. This conclusion is consistent with Leufkens et al. [43]. With the increase in the degree of matching between the construction land selected by the winning developer and the expected construction land, the greater the land degree of devaluation brought by the selection right to the loser developer, the greater the increase in the bidding difference between the winning and the losing developers in the second stage of construction land auction. This improves the transaction price of the construction land quota. Thus, proposition 5 is proven. Fig 3 also shows that the transaction price of construction land quota under the second-price sealed-bid sequential auction is significantly higher, which is contrary to the conclusion of Leufkens et al. [30]. This is because the private value assumed by Leufkens et al. [30] is certain. However, here, both the construction land quota and the construction land value are the developer’s private information. Thus, proposition 6 is proven. In addition, in the first-price sealed-bid sequential auction, since the quota transaction price is the developer’s highest quotation, the higher the value of the construction land quota to the developer, the higher the transaction price. In the second-price sealed-bid sequential auction, the quota transaction price is the developer’s second highest quotation. Therefore, with a higher value of construction land quota to the developer, the developers with the second highest quotation will increase. Consequently, the price of the quota will approach the real price. Therefore, the transaction price of the construction land quota first increases and finally tends to be flat.

### Analysis of farmers’ income

**Fig 4:**
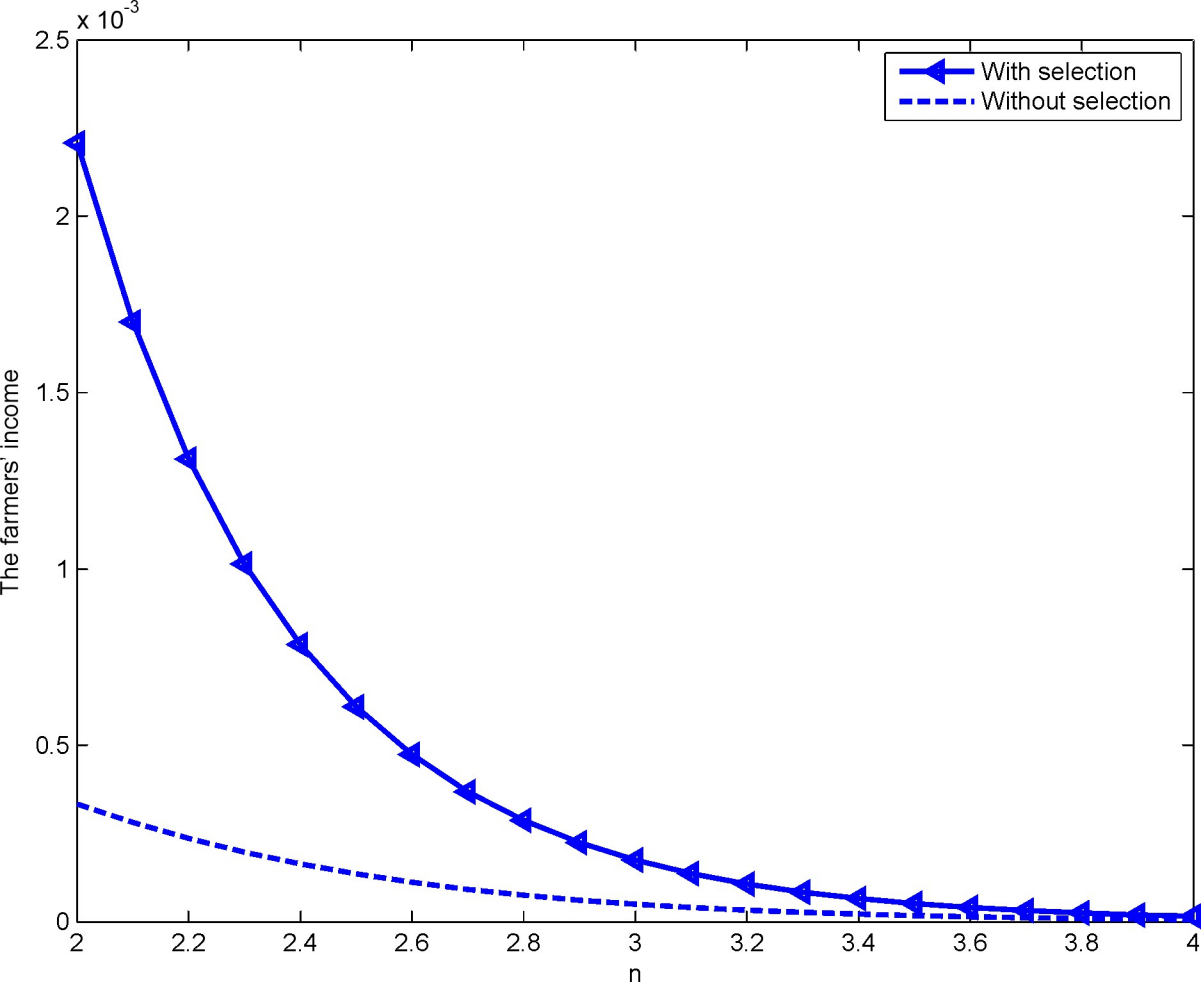
Impact of *n* on farmers’ income for the first-price sealed-bid format.

**Fig 5:**
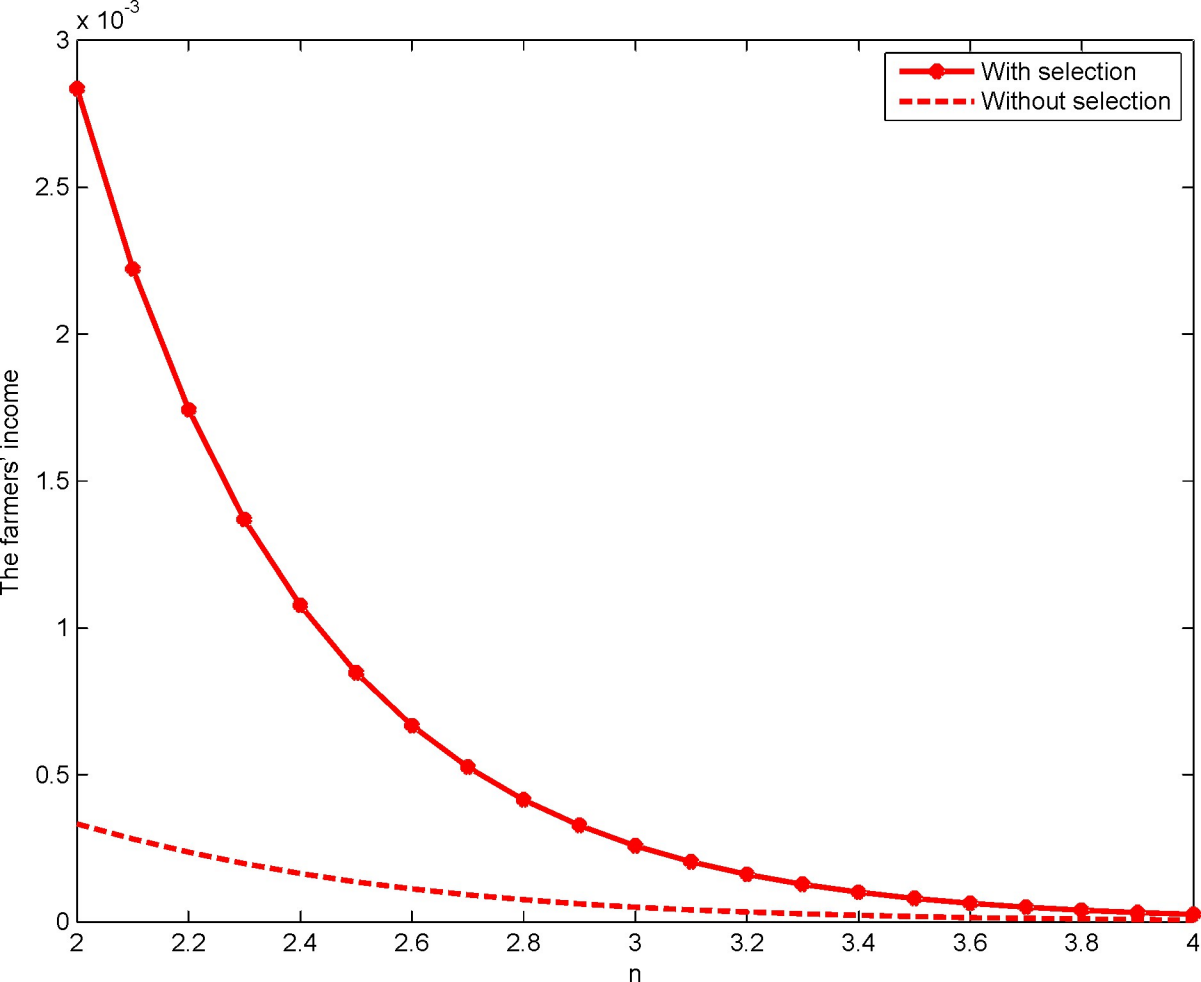
Impact of *n* on farmers’ income for the second-price sealed-bid format.

Using Matlab to simulate 10,000 auctions, we find that under the first-price (second-price) sealed-bid sequential auction with the right to choose, farmers’ income is the highest bid price (second highest quotation) of the developer. According to data from the Chongqing country land exchange, the quota transaction price of construction land is much smaller than the transaction price of construction land, approximately 0.1 times the price of construction land. Thus, let *ε* – 0.1. The relationship between selection rights and farmers’ income under first- and second-price sealed-bid sequential auctions are more clearly illustrated in Figs 4 and 5:

In Figs 4 and 5, the triangle and star lines represent farmers’ income when they have the right of selection under the first- and second-price sealed-bid sequential auction, respectively. The dashed line represents farmers’ income when there is no right of selection. Notably, in either auctions, farmers’ income is always significantly higher when there is a right of selection than when there is none, thus proving proposition 4. Meanwhile, with the increase in the number of developers participating in the auction, farmers’ income will decrease. This happens no matter whether they have the right of selection or not, thereby proving proposition 7.

**Fig 6:**
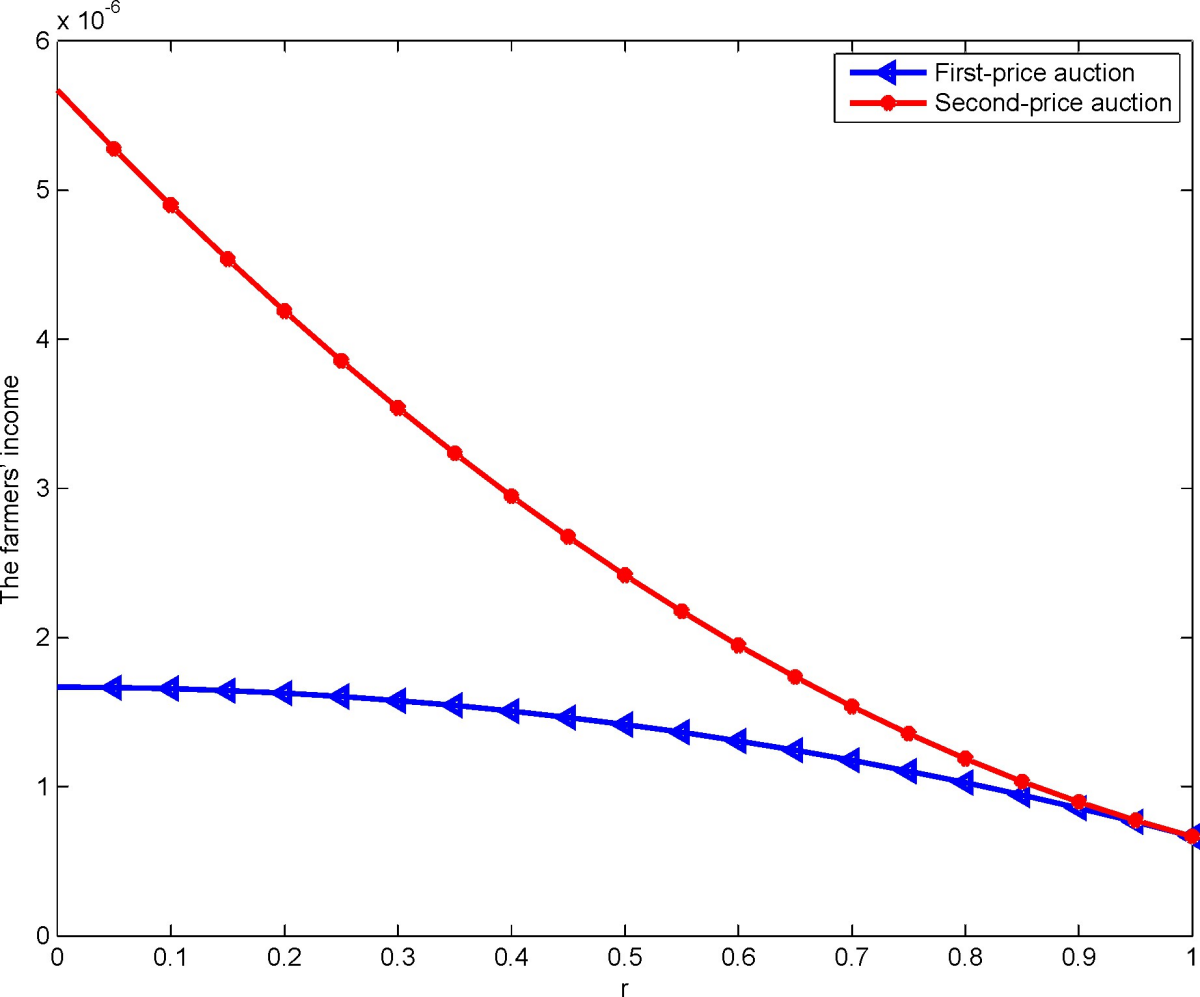
Impact of $r_{L,F_{j}}$ on farmers’ income.

The farmers’ income under the first- and second-price sealed-bid sequential auctions, and the change of farmers’ income with the land depreciation coefficient when there is the right of selection are shown in Fig 6:

Fig 6 shows that no matter the first- or second-price sealed-bid sequential auction, there is a negative correlation between the land depreciation coefficient and farmers’ income. That is, the higher the degree of matching between the construction land selected by the developer with the selection and the expected construction land, the greater the land depreciation of the construction land to the developer without the selection. This improves the value of the quota selection and farmers’ income. Thus, proposition 5 is proved. Meanwhile, the star line is clearly above the triangle line; that is, the revenue of the second-price sealed-bid sequential auction is significantly higher than that of the first-price one. This proposition extends the research hypothesis of Vickrey [44] and Myerson [45], and obtains the same result as Maskin and Riley [42], thus proving proposition 6. In the first-price sealed-bid sequential auction, farmers’ income is the highest bid of the developer for the construction land quota. Therefore, the higher the value of the construction land quota is for the developer, the more clearly farmers’ income increases. In the second-price sealed-bid sequential auction, farmers’ income is the second highest quotation of construction land quota from developers. Therefore, with the higher value of construction land quota to developers, the developers’ valuation of the second highest quotation will gradually approach the real price of construction land quota. Therefore, farmers’ income tends to be flat in the end.

## Conclusions

Based on the selection characteristics of construction land quota and the theory of sequential auction, this study constructs a model of construction land quota equilibrium bidding and farmers’ expected income under the first- and second-price sealed-bid sequential auction. Furthermore, we compare the equilibrium bidding strategy of bidders and farmers’ expected income under the two auction methods, and designs a more reasonable quota auction pricing mechanism. Finally, we use numerical analysis to verify the model and conclusions. The results show that the quota selection of construction land improves the bidding price of the quota and farmers’ income. Furthermore, the value of the selection to developers is positively correlated with the bidding quotation of construction land quota and farmers’ income. That is, the lower the degree of matching between the selected construction land and the developers without the selection, the higher the price developers are willing to pay for the quota and higher the farmers’ income. Besides, the second-price sealed-bid sequential auction has higher construction land quota bidding price and farmers’ income than the first-price auction. Third, there is a negative correlation between the number of developers participating in the auction, and the quota bidding quotation and farmers’ income.

These findings show that local governments can improve the value of construction land quotas. In the transaction practice of construction land and its quotas, developers can choose the expected land, thus obtaining more valuable quotas. This will improve the quotation of the quotas, ensure the income of farmers reclaiming the idle homestead, and improve the enthusiasm of farmers’ reclamation, thereby accelerating the process of land use for rural construction in China. Furthermore, choosing the second-price sealed-bid sequential sealed auction can better reflect the value of construction land quota, relieve the pressure of urban construction land demand, improve the exit rate of rural idle homesteads, realize land resource reallocation, and solve the contradiction between the rapid growth of construction land demand and the idle waste of a large number of rural homesteads. In addition, we should improve the qualification requirements of developers, increase the number of developers participating in the bidding, improve the pricing system of construction land quota, increase farmers’ reclamation income, narrow the gap between urban and rural areas, further help poverty alleviation and rural revitalization, and ensure the healthy and stable operation of construction land quota market.

Lastly, there are some limitations in this study and corresponding future research directions. The model used here studies a two-stage sequential auction and each stage only has a single item. However, in practice, there will be numerous homogeneous items auctioned at each stage. In the sequential auction of homogeneous items, there may be a decline in the prices of post-auction items and the phenomenon of "abnormal price decreases" [46]. This will impact the auction strategy of construction land quotas. These points require further research.
